# Disruption of testicular histoarchitecture and plasma hormone concentrations due to heat stress in donkeys

**DOI:** 10.3389/fvets.2026.1732107

**Published:** 2026-02-09

**Authors:** Muhammad Faheem Akhtar, Ejaz Ahmad, Ayman Abdel-Aziz Swelum, Qingshan Ma, Changfa Wang

**Affiliations:** 1 Research Institute of Donkey High-Efficiency Breeding and Ecological Feeding, College of Agronomy, Liaocheng University, Liaocheng, China; 2 Department of Clinical Sciences, Faculty of Veterinary Sciences, Bahauddin Zakariya University, Multan, Pakistan; 3 Department of Animal Production, College of Food and Agriculture Sciences, King Saud University, Riyadh, Saudi Arabia

**Keywords:** donkey, plasma hormone concentrations, short-term heat stress, spermatogenesis, testicular histoarchitecture

## Abstract

To maximize reproductive performance in farm animals, they must be protected from all kinds of stress, including heat stress (HS). Stress affects reproductive efficiency and the normal functioning of the hypothalamic–pituitary–gonadal (HPG) axis. The current study aimed to elucidate the effects of heat stress on reproductive efficiency in donkeys, including testicular histoarchitecture, stages of spermatogenesis, and variations in the plasma concentrations of testosterone, inhibin A, inhibin B, and luteinizing hormone (LH). A total of 30 Dezhou donkeys (Sanfen breed) were equally divided into two groups: Group A (*n* = 15) and Group B (*n* = 15). The animals in Group A were kept in an environmentally controlled shed where the temperature was maintained at 25 °C, while the animals in Group B were exposed to natural sunlight and kept in a barn during June, July, and August, when the average daily temperature ranged between 31 and 34 °C. Histological analysis revealed that HS decreased the seminiferous tubule diameter (STD), the number of spermatogonia, the number of seminiferous tubules (STs) per field, the number of spermatocytes, and the number of round and elongated spermatids. The diameter of the seminiferous tubules (STs) and epithelial height (EH) were positively correlated in June and August. In addition, STD, luminal tubular diameter (LTD), and the number of spermatocytes and round spermatids were positively correlated. In summary, we concluded that short-term heat stress reduces germ cells, spermatogenesis, and the number of spermatocytes and spermatids. It also causes alterations in testicular histoarchitecture without affecting the stages of spermatogenesis and decreases plasma concentrations of testosterone and luteinizing hormone. Moreover, inhibin B is the predominant form of circulating inhibin in donkeys.

## 1 Introduction

Heat stress (HS) exposure to animals not only reduces resistance to diseases but also lowers their growth and reproductive efficiency (1). In female animals, growing ovarian follicles are affected, ultimately impairing conception. In male animals, plasma hormones, spermatogenesis, and sperm quality are badly affected (1). Spermatogenesis is a highly complex mechanism involving mitosis and meiosis, and it requires coordination of germ cell differentiation (2). To achieve optimized animal production, managing heat stress is essential. The literature shows that environmental factors, including heat stress, have a deleterious effect on animal reproduction. For normal spermatogenesis, mammalian testes should be kept at 4–6 °C below internal body temperature (3). Managing heat stress is equally important for seasonal breeders, as they breed during a specific season. Ideal climatic conditions of air temperature and humidity have not yet been reported for Dezhou donkeys. From previous studies, it is evident that one spermatogenic cycle lasts for 10.5 days in donkeys and that 4.5 spermatogenic cycles complete the cycle of spermatogenesis in 47.2 days (3). Based on the acrosomal system, the seminiferous epithelium (SE) in donkeys can be divided into 12 stages, which are further classified into undifferentiated spermatogonia (A_und_) and differentiated (A_1_, A_2_, A_3_, B_1,_ and B_2_) spermatogonia (4). Germ cells near the basal membrane of the seminiferous epithelium (spermatogonia) are more vulnerable to heat stress due to their intense cell division activity (5). In rats, humans, and bovines, younger spermatids and pachytene spermatocytes are the most susceptible to hyperthermia (6, 7). The duration of spermatogenesis in donkeys (*Equus asinus*) is 42.7 days, and the entire process of spermatogenesis takes 4.5 cycles (each cycle lasts for 10.5 days), which are further divided into 12 stages, including undifferentiated spermatogonia (Aund) and differentiated spermatogonia (A1, A2, A3, B1, and B2) (8).

In mammals, germ cells are strategically positioned within the seminiferous epithelium in a well-organized cyclic manner, forming specific groupings known as stages. The seminiferous epithelium cycle involves a series of events that occur between these stages. This process includes the disappearance of a particular cellular arrangement, followed by its reappearance in a designated region of the seminiferous epithelium. These cell groupings can be identified using two methods: (a) by evaluating the morphological traits of spermatids and the overall alignment of germ cells and (b) by monitoring the development of the acrosomal system (9). Regarding the impact of heat stress (HS) on reproductive function in mammals, spermatogenesis is particularly susceptible, often resulting in varying degrees of reduced fertility (10). Regional variations in the extent of ambient temperature increase are significant, and there are also notable differences among species and individuals in their sensitivity to heat stress (11). High temperatures can substantially impact various aspects of reproductive processes in mammals (10). These effects include disruptions in sperm and egg formation, impaired egg maturation, compromised early embryonic development, altered fetal and placental growth, and reduced lactation. These harmful consequences of heat stress arise either from the elevated levels of body temperature or from the physiological adjustments that heat-stressed animals make to maintain their body temperature. All germ cells are sensitive to heat stress, including Leydig and Sertoli cells (12), but the most vulnerable are pachytene spermatocytes, spermatids, and epididymal sperm (13–15). The effects of elevated testicular temperature depend on the magnitude and duration of heat exposure. While a short-term, mild increase in testicular temperature may cause only a temporary decline in sperm quality, continuous or intense heating is more likely to lead to infertility (16). Additionally, prolonged and severe thermal injury could halt spermatogenesis permanently.

Although a substantial body of literature documents the impacts of heat stress in female animals, studies on male animals—especially donkeys—remain limited. Therefore, the purpose of the present study was to evaluate the effects of heat stress during June, July, and August on testicular histoarchitecture, including alterations in seminiferous epithelium dimensions, Jonhson’s score, stages of spermatogenesis, and testes weight, as well as plasma concentrations of testosterone, luteinizing hormone (LH), inhibin A, and inhibin B. This investigation will contribute to understanding the effects of short-term heat stress on reproductive physiology and spermatogenesis in Dezhou donkeys.

## 2 Materials and methods

### 2.1 Ethical statement

The experimental animals and methods used in this study were approved by the Animal Policy and Welfare Committee of Liaocheng University (No. LC2019-1). The care and use of laboratory animals fully comply with local animal welfare laws, guidelines, and policies.

### 2.2 Animals and farm location

The current study was conducted in Liaocheng (western Shandong province) at the Don E Jiao donkey farm (a government-owned donkey breeding company), located at 36° N and 115° E, at an elevation of 37.54 m (123.16 feet) above sea level. This study was conducted in June, July, and August. The average temperature ranged from 30 to 31.11 °C, and the annual rainfall ranged from 76.4 to 153.7 mm. (17). A total of 30 (*n* = 30) adult Dezhou donkeys (*Equus asinus*, Sanfen breed), aged 4–6 years and weighing 260 + 12 kg, all of the same genetic origin, were equally divided into two groups: Group A and Group B. Each group had 15 animals (*n* = 15). The donkeys in Group A were kept in an environmentally controlled shed, where the optimum room temperature was 25 °C, and the group served as the experimental group. The animals in Group B were kept in an outside shed, where the room temperature ranged from 30 to 31.11 °C from June to August. This group served as the heat-stressed group. All animals had *ad libitum* access to the mineral mixture and drinking water.

### 2.3 Testes and plasma sample collection

Five animals from each group were slaughtered on 30 June, 30 August, and 30 July for the collection of testes and blood samples. Blood samples were collected via jugular venipuncture into heparin tubes before slaughtering the donkey at the slaughterhouse. Plasma was separated from the blood samples within 3 h of collection by centrifugation at 1,000 g and stored at 20 °C until analysis.

### 2.4 Measurement of body weight and testicular weight

The body weight of each Dezhou donkey in Groups A and B was measured at the end of June, July, and August. Five donkeys from Groups A and B were taken to the slaughterhouse for the collection of testicular samples. The combined weight of both testes from each animal was measured. Immediately after the collection, testicular tissues were immersed in a 10% formalin solution for further analysis.

### 2.5 Microscopy performance

A 0.125 cm^3^ sample of the left testicular tissue was collected and embedded in a 10% neutral buffered formalin solution for 24 h to examine histological alterations in the seminiferous tubules (STs). Histological analysis was performed using an automated tissue processor (LECIA RM 2235). After fixation, the tissues were dehydrated through a graded series of ethanol solutions (70, 80, 90, 100%, and absolute alcohol). After dehydration, the tissues were cleared in xylene and embedded in paraffin wax. Testis tissues were cut perpendicular in 5 μm thickness to testicular long axis. Slides were then mounted on glass slides and were stained using hematoxylin and eosin (Nanjing Jiancheng Bioengineering Institute, Nanjing, China). Various histomorphometric parameters of the seminiferous epithelium were measured, including the diameter of the seminiferous tubules (ST), ST/field, luminal tubular diameter (LTD), and the numbers of spermatogonia, spermatocytes, and elongated spermatids. Moreover, epithelial height was determined as described previously (18). All STs were examined under a bright-field light microscope (LEICA Dmi8, Germany) at 10× (100 μm), 20× (75 μm), and 40× (25 μm) magnifications. Johnson’s score was used to assess germ cell development, with scores ranging from 1 to 10 according to the following criteria:

10 = complete spermatogenesis;
9 = spermatozoa present with random spermatogenesis;
8 = few spermatozoa;
7 = no spermatozoa, but spermatids present;
6 = few spermatids present;
5 = only spermatocytes present;
4 = few spermatocytes present;
3 = spermatogonia present;
2 = only Sertoli cells present; and.
1 = lumen almost empty.

The mean score was calculated by randomly selecting 10 STs per animal.

### 2.6 Spermatogonial kinetics

The stages of the seminiferous epithelium were characterized based on the acrosomal system, the morphological development of spermatid nuclei, and the association of germ cells (19, 20). A total of 10 seminiferous tubules/animals were examined at 40× (25 μm) magnification to determine relative stage frequencies. Various spermatogonial types were classified by analyzing the seminiferous epithelium cycle in donkeys (*Equus asinus*) (4). Germ cell differentiation was assessed based on nuclear shape, heterochromatin presence, and nucleolar compaction (21, 22). Spermatogonia were classified according to their morphological features at each stage of the seminiferous epithelium. The nuclear volume of each spermatogonial type was analyzed by measuring the diameter of 20 nuclei per animal (21). Spermatogonial cell types were counted at each stage of the seminiferous epithelium and expressed as the number of spermatogonia per 1,000 Sertoli cell nuclei to determine spermatogonial kinetics (23). In Dezhou donkeys, spermatogonial morphology was consistent with previously described patterns (8).

### 2.7 Seminiferous epithelium cycle evaluation using the acrosomal system

Stages of spermatogenesis in Dezhou donkeys were evaluated based on the spermatid acrosomal system and the association patterns of germ cells. Alteration in germ cells was identified according to the acrosomal system in mice and rats. These changes included coalescence, vesicle formation, nuclear capping, and elongation of spermatids (9). Following the analysis of the acrosomal system, various germ cells (including spermatogonia, spermatocytes, and spermatids) were identified according to their morphological stage. The stages of the seminiferous epithelium were determined using two previously described approaches (4). From each jack, 100 seminiferous tubule cross-sections were identified for the relative frequencies of the seminiferous epithelium stages.

### 2.8 Plasma concentrations of inhibin a, inhibin B, testosterone (T), and LH

Plasma concentrations of testosterone, LH, inhibin A, and inhibin B were measured by ELISA using the Quantitative Diagnostic Kit for testosterone (North Institute of Biological Technology, Beijing, China). Plasma concentrations of inhibin A and inhibin B were determined using commercial ELISA kits (AL-161: Equine–canine–rodent inhibin-A ELISA and AL-163: Equine–canine–rodent inhibin-B ELISA; Ansh Laboratories, Webster, TX), which have been previously validated for equine samples (24). The intra-assay coefficients of variation for inhibin A and inhibin B were 1.6 and 8.2%, respectively. Plasma testosterone and LH were determined by RlA, as previously reported (31). Intra-assay and inter-assay CVs were 9.9 and 11.5% for testosterone and 8.8 and 9.5%. LH was determined by RlA, as previously reported (25). For testosterone, Intra-assay and inter-assay CVs were 9.6 and 11.3%. Plasma concentrations of testosterone were determined by ELISA using the Quantitative Diagnostic Kit for testosterone (North Institute of Biological Technology, Beijing, China).

### 2.9 Statistical analysis

Normality of data distribution was assessed using the Shapiro–Wilk test, and homogeneity of variances was evaluated using Levene’s test. Based on these results, appropriate statistical tests were selected. When assumptions were met, one-way ANOVA was used to assess differences between the two groups for each month, followed by Bonferroni *post-hoc* tests. When assumptions were violated, appropriate non-parametric tests were used. A *p* < 0.05 was considered statistically significant. GraphPad Prism (version 5.0, California, United States) and SPSS (version 20.0, New York, United States) were used for statistical analysis.

## 3 Results

### 3.1 Body weight, testes weight, and the testes-to-body weight ratio

**Figure 1:**
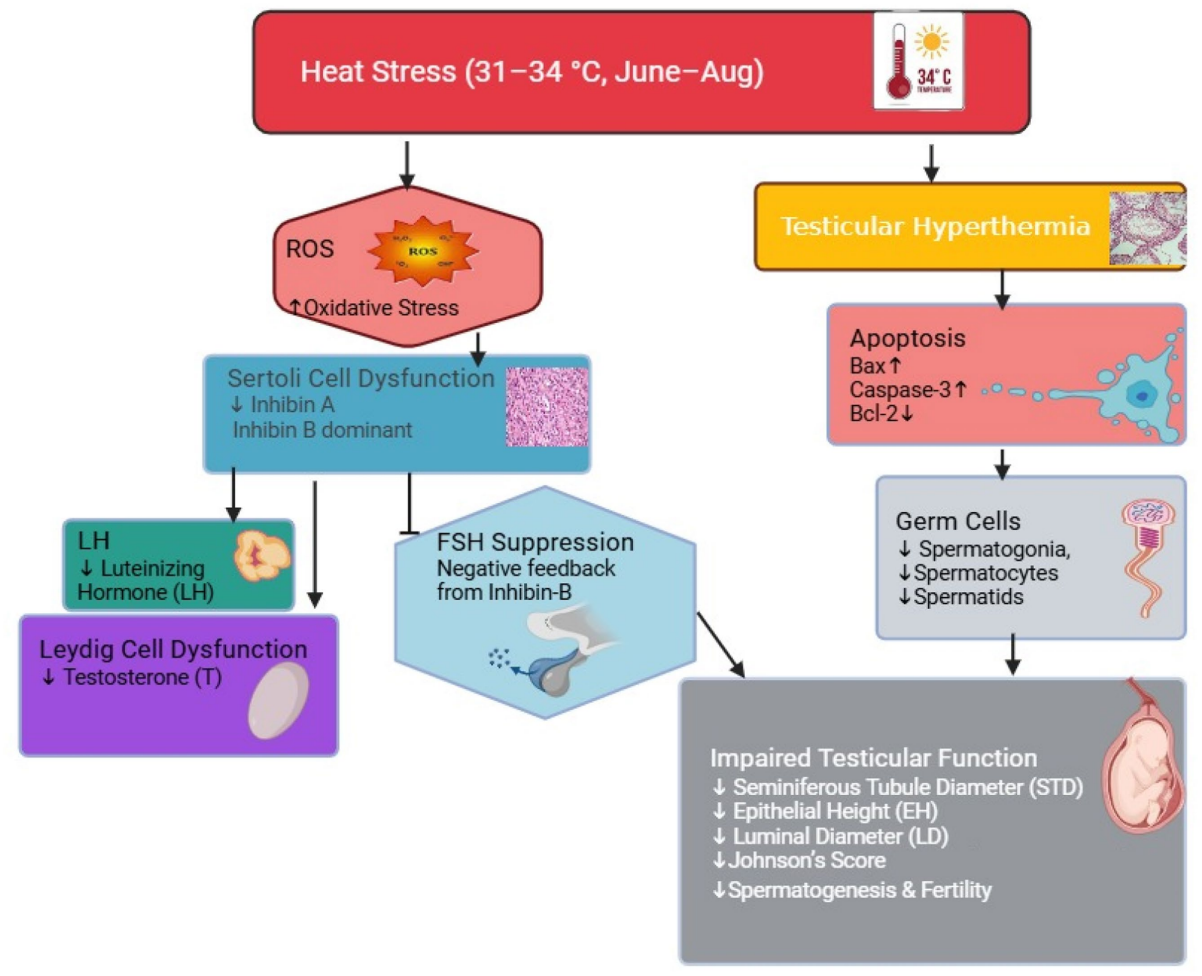
Detrimental effects of heat stress on donkey’s testicular histoarchitecture. Heat stress impairs spermatogenesis, impairs Sertoli cell function, and Leydig cell function, lowering FSH and fertility.

Body weight showed non-significant differences (*p* > 0.05) between Groups A and B, as shown in Figure 1A. Testes weights also exhibited non-significant differences throughout the experimental period between the groups, although they were slightly higher in the control group (Group A) at the end of July and August (Figure 1B). The testes-to-body weight ratio also showed a non-significant difference (Figure 1C).

### 3.2 Diameter of ST, LD, EH, number of ST/field, and germ cells

**Figure 2:**
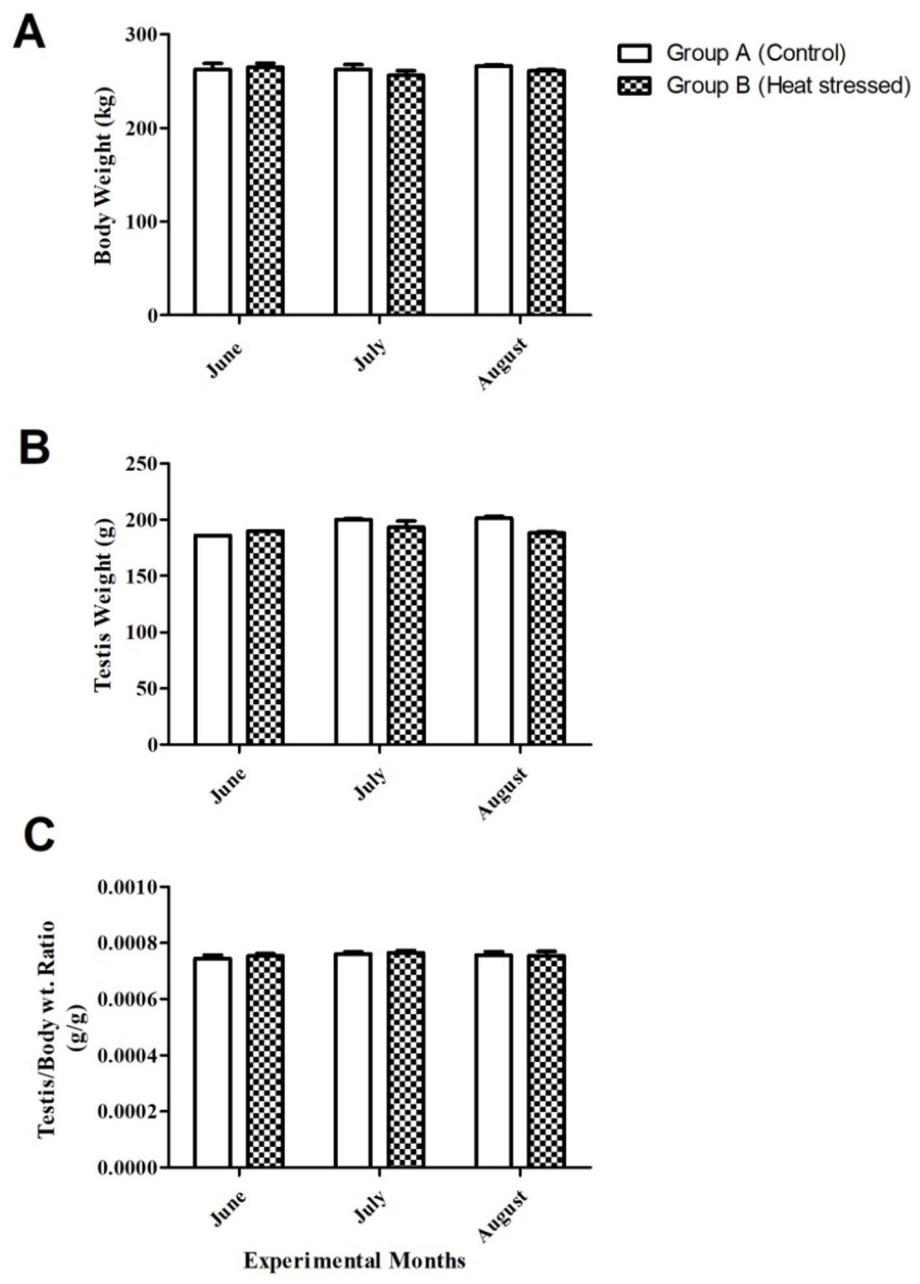
**(A)** Body weight, **(B)** testis weight, and **(C)** testis to body weight ratio in groups A (control) and B (experiment group) of Dezhou donkeys.

**Figure 3:**
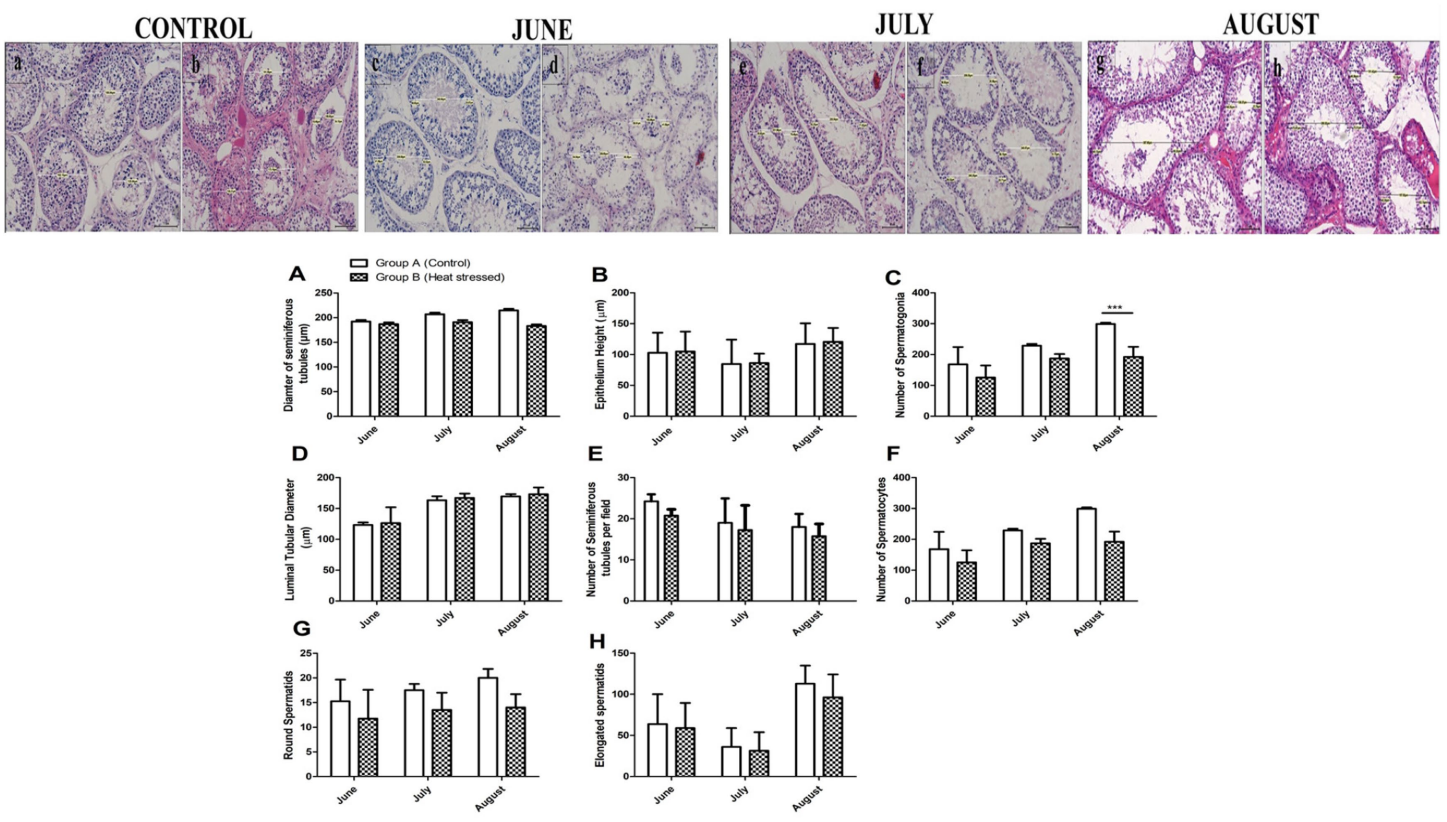
Determination of histological measurements of seminiferous tubules in Dezhou donkeys. TD-tubular diameter and LD-luminal tubular diameter determined by the difference between TD and the sum of epithelial heights (EH1+EH2). Epithelial height determined by means of EH1 and EH2, with a scale bar of 75μm at 20X magnification. **(a,b)** Histological diagrams of the control group. **(c–h)** Histological measurements of seminiferous tubules in June, July, and August, respectively. **(A–H)** Morphometric measurements of histological sections and germ cell numbers. Data are shown as the mean values ± standard error of the mean. ** and *** indicate statistical significance based on *p* < 0.01 and *p* < 0.001, respectively, between groups A (control) and B (short-term heat-stressed). Each group has *n* = 4.

The histological diagram in Figure 2 illustrates the method used for measuring ST diameter, epithelial heights (EH1, EH2), and luminal tubular diameter (LD) in testicular histoarchitecture. Figure 2 also presents a graphical summary of histological parameters, including ST diameter (μm), ST/field, epithelial height (μm), luminal tubular diameter (μm), and numbers of elongated spermatids, spermatocytes, spermatids, and spermatogonia. A positive correlation was observed between ST diameter, EH, luminal tubular diameter, and germ cell numbers in all animals. As depicted in the histological diagrams (Figures 3A–D), the number of germ cells in the heat-stressed group (Group B) decreased progressively in June, July, and August. Similarly, the number of germ cells, including spermatogonia, spermatocytes, and round and elongated spermatids, was higher in Group A than in Group B throughout the experimental period.

### 3.3 Kinetics and morphological features of spermatogonial types

According to the criteria described in Chiarini-Garcia et al. (4), spermatogonial cells in Dezhou donkeys were classified as undifferentiated (A_und_) and differentiated (A_1_, A_2_, A_3_, B_1_, and B_2_), as depicted in Figure 3. Morphological features of Aund spermatogonia included a spherical nucleus with small granules and minimal heterochromatin. Granular euchromatin was observed in A_1_, A_2_, and A_3_ spermatogonia, with heterochromatin content increasing progressively from A_1_ to A_3_. Spermatogonia B_1_ and B_2_ exhibited an egg-shaped nucleus, and the nucleoplasm seemed granular. Various differentiated spermatogonia were also observed in the seminiferous epithelium cycle, along with A_und_ spermatogonia. Morphological analysis of the seminiferous epithelium cycle revealed the presence of A1 spermatogonia in stages VII, VIII, and IX; A2 spermatogonia in stages X and XI; and A3 spermatogonia in stages XII and I. B1 and B2 spermatogonia were observed in stages II, III, IV, V, and VII of the seminiferous epithelium, respectively. Among the differentiated spermatogonia, cell numbers increased from A1 to B2, while A3 to B1 spermatogonia showed a non-significant decline.

### 3.4 Stages of the seminiferous epithelium cycle

Seminiferous epithelium (SE) stages were characterized to determine whether temporary heat stress affects spermatogenesis. Examination of the SE stages indicated that the morphological development of germ cells was normal in the animals from the control group. In contrast, the development of spermatogonia, spermatocytes, and spermatids was affected in the experimental group, leading to signs of apoptosis due to the higher temperature. All 12 stages of the seminiferous epithelium (I–XII) were observed, as shown in Figure 3. The effects of high environmental temperature on the seminiferous epithelium stages in the experimental groups are shown in Table 1.

**Table 1:** Presence of various germ cells during stages of the seminiferous epithelium cycle in Dezhou donkey.

Stages	Spermatogonia	Pachytene spermatocytes	Spermatids
I	A_und_, A_3_, B_1_	Small	Steps 1 and 13
II-III	A_und,_ B_1_	Small	Steps 2 and 3, 14 and 15
IV	A_und,_ B_1_	Larger	Steps 4 and 15
V	A_und,_ B_1_, B_2_ (few)	Moved to the middle of the seminiferous epithelium	

### 3.5 Johnson’s score

**Figure 4:**
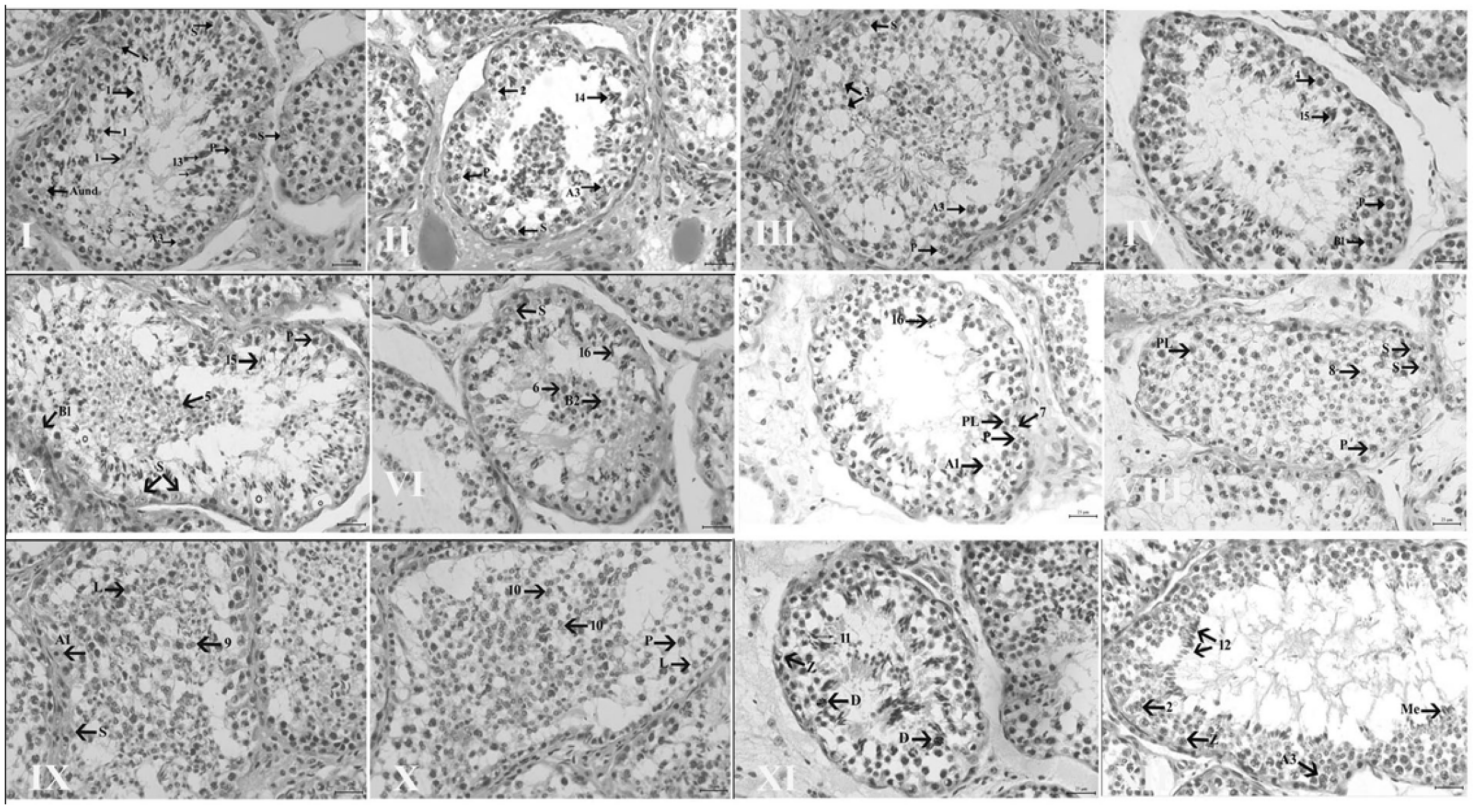
High-resolution light photomicrographs of the XII stages of the donkey’s seminiferous epithelium cycle, characterized according to the development of the acrosome. Roman numbers indicate the stages of the seminiferous epithelium cycle: Aund, type A undifferentiated spermatogonia; A1, A3, B1, and B2, differentiated spermatogonia; Pl, preleptotene; L, leptotene; Z, zygotene; P, pachytene; D, diplotene; and Me, meiotic. All images were observed at 75 μm at 20 ×.

Johnson’s score was assessed according to the criteria described in section 2.3. Figures 4A–F shows Johnson’s score ranging from 1 to 8. For Johnson’s score assessment, 60 images were captured from each animal, and one image was chosen as representative. Figures 4A,C,E show Johnson’s score in the control group, indicating normal spermatogenesis. At the end of June, the score was 8 in Group A, while it was 7 in the heat-stressed group. One month later, at the end of July, the score in the experimental group was 5, while it was 4 in the heat-stressed group. It continued to decrease in August and reached its lowest level in the heat-stressed group. Figure 4F shows a complete absence of spermatogenesis.

### 3.6 Seasonal fluctuations in plasma hormone concentrations (INH-A, INH-B, T, and LH)

**Figure 5:**
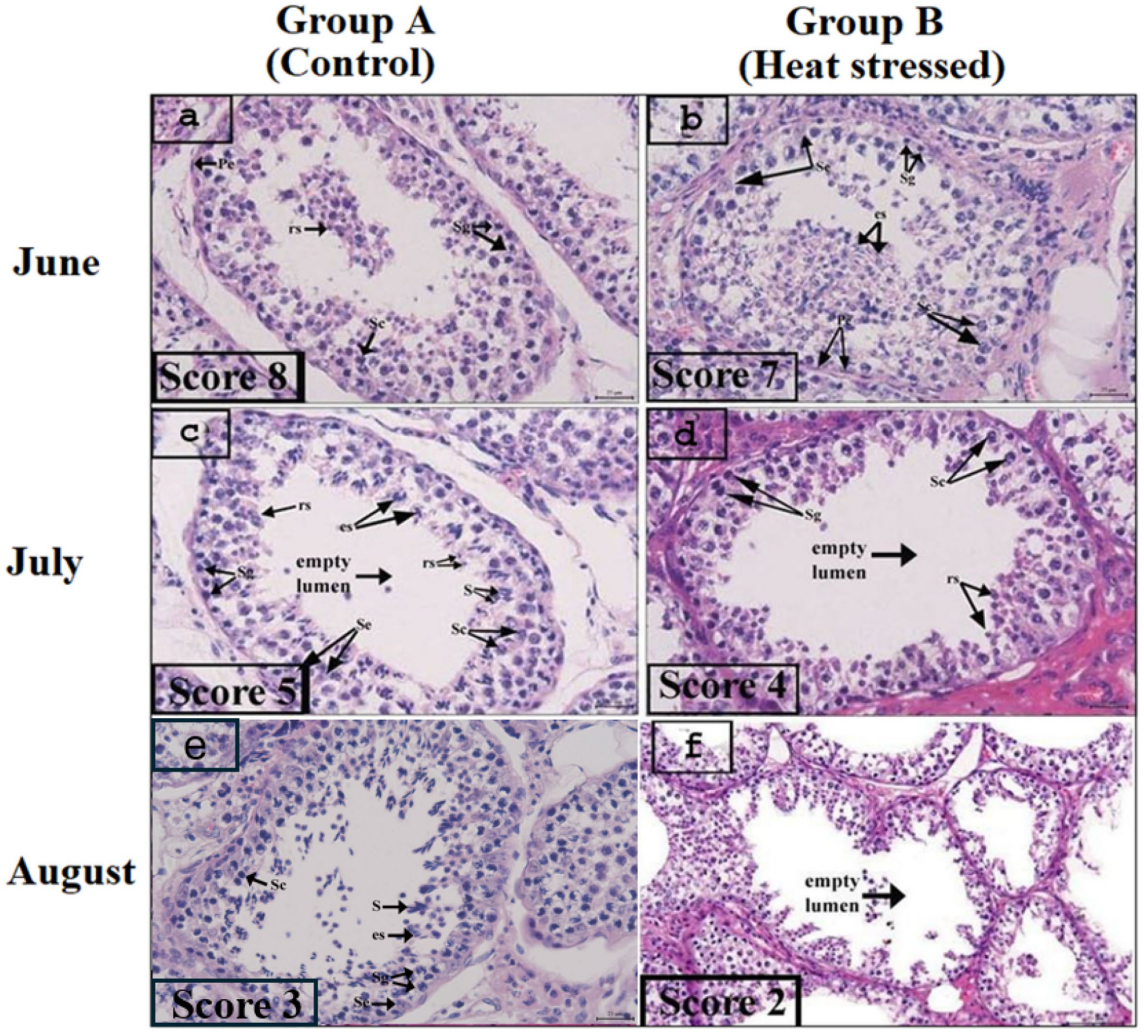
**(a–h)** Depict Johnson’s score. **(a,c,e)** Depict Johnson’s score in the experiment, and **(b,d,f)** Johnson’s score in heat stressed group. Le, Leydig cells; S, spermatozoa; es, elongating spermatids; rs, round spermatids; Sc, spermatocytes; Sg, spermatogonia; and Se, Sertoli cells. All images were observed at 75 μm at 20 ×.

**Figure 6:**
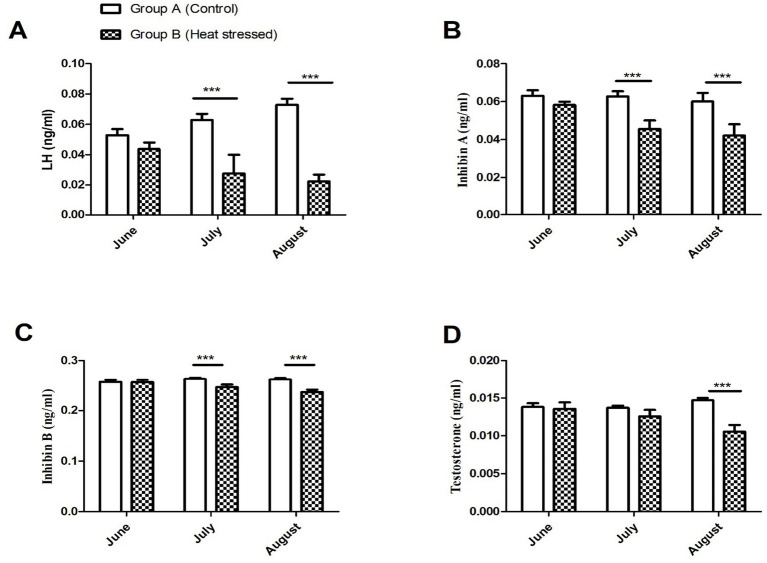
**(A–D)** Plasma hormone concentrations of luteinizing hormone (LH), Inhibin-A, Inhibin-B, and testosterone in groups A (control) and B (experimental group) during June, July, and August. Data are shown as mean values ± standard error of the mean. *, **, and *** indicate difference at *P* < 0.05, *P* < 0.1, and *P* < 0.001, respectively, between the groups.

Figure 5 illustrates the plasma concentrations of LH, inhibin A, inhibin B, and testosterone in June, July, and August in Groups A and B. Plasma luteinizing hormone (LH) levels remained relatively stable in Group A throughout the experimental period compared to Group B, as depicted in Figure 4A. Initially, the LH level was 0.05 ng/mL in the control group (Group A), while, at the same time, it was 0.04 ng/mL in the experimental group (Group B). In July, LH levels (ng/mL) in Group A continued an ascending pattern and reached 0.065 ng/mL, while in the experimental group, LH decreased to 0.03 ng/mL. There existed a significant difference (*p* < 0.001) in July among the two groups. Similar patterns were observed in August (*p* < 0.001). Plasma inhibin A (ng/mL) remained higher in the control group (Group A) throughout the experimental period compared to the heat-stressed group (Group B). In Group A, the level was 0.06 ng/mL initially and remained nearly constant in July and August, while it showed a declining pattern in the experimental group (Group B). Plasma inhibin A was significantly higher (*p* < 0.001) in Group A than in Group B during July and August. In July, the concentrations were 0.061 ng/mL in Group A and 0.041 ng/mL in Group B, while in August, they were 0.062 ng/mL and 0.040 ng/mL, respectively. Plasma inhibin B was significantly higher (*p* < 0.001) in group A compared to group B in July and August. Testosterone levels also remained higher in the control group, while in the experimental group, they showed a declining pattern. A significant difference (*p* < 0.001) in plasma testosterone (T) concentrations was observed in August, with values of 0.015 ng/mL in the control group and 0.010 ng/mL in the experimental group (Figure 6).

## 4 Discussion

Heat stress is a major challenge for livestock. It not only compromises animal welfare but also causes health and productivity losses. To the best of our knowledge, this is the first study to investigate the effects of short-term heat stress on donkeys. Hyperthermia has detrimental effects on testosterone and inhibits spermatogenesis in mine, horse, sheep, cows, and pigs (26–31). Although the effects of short-term heat stress are well documented in other mammalian species, studies in donkeys are lacking. The process of spermatogonial differentiation into sperm is highly complex, and various factors, such as hormones, growth factors, seasonality, and testicular temperature, regulate it (32, 33). Our results are consistent with previous studies showing that heat stress induces oxidative stress, leading to apoptosis and damaged germ cells. Elevated testicular temperature disrupts the normal thermoregulatory mechanism of the testes and induces oxidative stress in germ cells, ultimately reducing sperm function and resulting in male infertility (34, 35). Normal development of spermatogonia, spermatocytes, and spermatids is essential for achieving high fertility, optimal semen quality, and spermatogenesis (36). Heat stress leads to apoptosis and germ cell autophagy, which can ultimately damage sperm DNA and increase the production of reactive oxygen species (ROS) (37). In mice, exposure to heat stress reduced the ability of sperm to fuse with oocytes both *in vivo* and *in vitro* (38). The hot season damaged the seminiferous epithelium in rams, leading to abnormal and non-viable sperm during mid-summer (39). Early stages of spermatogenesis were affected by heat stress in rams (30). This evidence suggests that heat stress induces oxidative stress in the testes, leading to germ cell apoptosis and ultimately lowering donkeys’ semen quality. The lower number of germ cells in the heat-stressed group suggests apoptosis in the seminiferous epithelium.

Body weight, testicular weight, and the testes-to-body weight ratio showed no significant differences and a similar ascending and descending order in all animals, as shown in Figure 1. These results indicate that short-term heat stress has no effect on testicular morphology. Mariane et al. (40) concluded that stallions are seasonal breeders, but seasonality in reproduction depends on the latitude of origin; alterations in seasonality affect gonadal activity in stallions (40). Our findings align with those of Freitas et al. (40), who concluded that seasonality and higher altitudes affect variation in reproduction. It may be one of the reasons why the hot months did not affect testicular weight. Figure 2 depicts the normal progression of spermatogenesis and the normal development of germ cells in all seminiferous tubules, including spermatogonia, spermatocytes, and spermatids.

The acrosomal system is considered the most effective method for analyzing various morphological alterations in the seminiferous epithelium. The accuracy of this method depends on stages and species [for example, six stages in humans (41) and 14 stages in rats (42)]. In previous studies, a tubular system has been applied to equine and asinine subjects. In the study by Chiarini-Garcia et al. (4), only the acrosomal method was used to analyze pre- and post-meiotic stage frequencies (~27%; stages VIII–XI, and ~55%; stages I–VII). Comparisons with the tubular morphological method showed similar results (8, 43, 44). In the present study, for the first time, the acrosomal system was combined with Johnson’s score, seminiferous tubule (ST) diameter, and germ cell quantification to evaluate alterations in the seminiferous epithelium of donkeys exposed to short-term heat stress. Our findings support previous observations that, within the same mammalian family, pre- and post-meiotic stage frequencies can be phylogenetically determined (45, 46).

Examination of spermatogenesis allowed us to distinguish distinct groups of spermatogonia based on their appearance. Using high-resolution light microscopy, we observed variations in nuclear morphology, allowing the separation of the earliest spermatogonia into two distinct categories: undifferentiated type A (A_und_) and differentiated type A spermatogonia. The morphological characteristics distinguishing these two groups closely resembled those observed in mice (22), rats (47), and golden hamsters (48). However, unlike observations in other species, the chromatin in donkeys appeared coarse in all animals examined in this study. This discrepancy may have resulted from the method of cell preservation, such as fixation, or other tissue-processing factors. Nonetheless, we observed subtle morphological features in neighboring cells, including spermatocytes, spermatids, and, especially, Sertoli cells.

In 1991, Johnson classified horse spermatogonia into five subtypes: A1, A2, A3, B1, and B2. However, Aund spermatogonia are similar to those found in rodents, as described by previous studies (48–50). As shown in Figure 2, we observed A_und_ spermatogonia in the seminiferous epithelium across all 12 stages of the seminiferous epithelial cycle, mirroring the kinetics reported in rodents, which have been extensively studied (51). As shown in Figure 2, the Aund spermatogonial population reached its lowest level during stage VIII of the seminiferous epithelial cycle. Subsequently, their numbers gradually increased, peaked at stage VI of the following cycle, and declined again at stage VIII. Swierstra et al. (44) observed the same trend in type A spermatogonial kinetics during the cell cycle in horses. A possible explanation for this kinetic pattern is that, during stages VII and VIII, A_und_ spermatogonia differentiate into A_1_ spermatogonia, giving rise to a new wave of spermatogonial differentiation, with a consequent reduction in the number of Aund spermatogonia, as described previously in rodents (52, 53). Our combined morphologic and stereological analyses allowed us to distinguish three types of differentiated spermatogonia (A1, A2, and A3) and two types of B spermatogonia (B1 and B2) in Dezhou donkeys. These cell types displayed distinct morphology under high-resolution light microscopy, and the kinetics along the cycle, from A_1_ to B2, showed that cell numbers increased progressively. Each spermatogonial type was observed in the stage where it originated via mitosis from the predecessor cell and in the next one (or two) stages when the spermatogonial type would rise by mitosis of the next spermatogonial type, as observed in mice (52) and golden hamsters (48). Therefore, it was also demonstrated in Dezhou donkeys that spermatogonial types overlap in their kinetics across the seminiferous epithelial cycle. In stage VIII, a further reduction was observed, mirroring the pattern observed in stallions by Swierstra et al. (44) for type A spermatogonial kinetics during the cell cycle. This trend may be explained by the differentiation of type A undifferentiated spermatogonia into type A1 spermatogonia during stages VII and VIII, initiating a new wave of spermatogonial differentiation and consequently reducing the number of type A undifferentiated spermatogonia, as previously documented in rodents (52).

Figure 2 shows a positive correlation between STD, EH, and LTD. It is widely accepted that there is a positive correlation between ST diameter, EH, testicular weight, and the process of spermatogenesis (18, 36). Similarly, a positive correlation was observed between ST/field and germ cell numbers (spermatogonia, spermatocytes, and spermatids). A positive correlation among these parameters indicates the normal progression of spermatogenesis and the development of germ cells within the testicular histoarchitecture. As previously reported, similar findings were observed in Yanghou ganders (18, 36). Despite differences between species, the principle remains the same for testicular histoarchitecture. As the experiment progressed, animals in Group A showed normal germ cell development and consistently exhibited slightly higher values at all stages compared to the experimental group. Based on testicular histoarchitecture and germ cell development, we can speculate that short-term heat stress reduces the number of germ cells through apoptosis, whereas normal temperature helps maintain normal testicular histoarchitecture.

Endocrinological changes after temporary heat stress in jacks are poorly understood. LH showed an ascending pattern in the control group (Group A), while there existed a descending pattern in the experimental group (Group B), as shown in Figure 4A. In July and August, plasma hormone concentrations in the control group were significantly higher than those in the experimental group. Similar reductions in plasma LH due to heat stress have been reported in sheep (54), cattle (55), and goats (56). Heat stress has been shown to reduce plasma LH in horses (57), lactating cows (58), and working horses (59). Our findings are consistent with previous observations reported by researchers. In most mammalian species, inhibin B is the predominant form regulating reproduction, except in rams, where inhibin A is more important (60). Inhibin A and inhibin B showed variations during the experimental period. Inhibin B is the predominant form of inhibin involved in male reproductive function compared to inhibin A (60). It is a major form of circulating inhibin in hamsters, miniature pigs, and rats (61, 62, 63). These findings, together with previously reported data in mares, indicate a sexually dimorphic pattern of inhibin isoform expression in horses, with inhibin B being more prevalent in stallions and inhibin A being more prevalent in mares (64). Our findings are in accordance with previous research. Inhibin B concentrations were four times higher than inhibin A throughout the experimental period. This study suggests that inhibin B exerts negative feedback on follicle-stimulating hormone (FSH). It can be speculated that FSH secretion is suppressed by inhibin B and that weak signaling in the heat-stressed group affected Sertoli cell function, resulting in reduced testicular weights. The consistently lower plasma testosterone concentrations observed in the experimental group compared to the control group can be attributed to heat stress-induced loss of Leydig cells and germ cells, leading to reduced testosterone (T) levels. Testosterone plays a vital role in the progression of spermatogenesis in the seminiferous tubules (65). The plasma testosterone concentration was significantly higher in the control group at the end of August. These findings highlight that even short-term heat stress reduces plasma testosterone concentrations in donkeys. Disrupted spermatogenesis and lower Jonson’s score are closely associated with the lower testosterone concentrations observed in the experimental group. Additional research is required to explore the molecular mechanisms underlying apoptosis after short-term heat stress, focusing on genes in germ, Sertoli, and Leydig cells and their effects on semen in donkeys.

## 5 Conclusion

The present study, conducted on donkeys, shows that temporary heat stress is a type of stress that affects testicular histoarchitecture, the seminiferous epithelium, and plasma concentrations of LH, inhibin A, inhibin B, and testosterone. Temporary heat stress affects Sertoli and Leydig cells without altering the stages of spermatogenesis. Inhibin B is the primary circulating form of inhibin in Dezhou donkeys. Maintaining an optimal temperature of 25 °C can help prevent these harmful effects during the summer months in commercial donkey farming, while also considering the economic factors of the enterprise. There are some limitations in our study. Daily temperature and humidity were not recorded. In addition, the experimental duration was limited due to funding constraints. These factors should be addressed in future research.

## Data Availability

The raw data supporting the conclusions of this article will be made available by the authors, without undue reservation.
